# Supplementary material for: SARS-CoV-2 neutralizing antibody specificities differ dramatically between recently infected infants and immune-imprinted individuals
Source: J Virol. 2025 Mar 25;99(4):e00109-25. doi: 10.1128/jvi.00109-25 (PMC11998527; doi:10.1128/jvi.00109-25)
Supplement: Supplemental material — Figures S1 to S9 and Table S1. [file jvi.00109-25-s0001.pdf]

Supplementary Figures

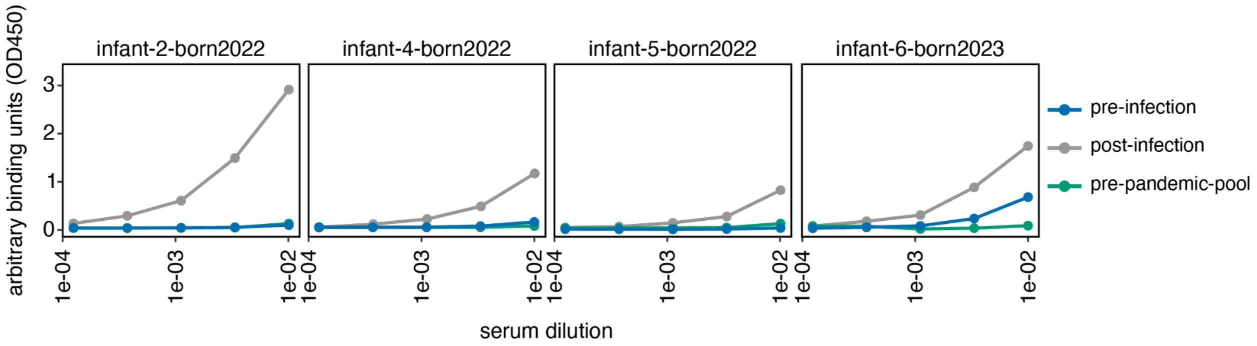

Supplementary Figure 1. Binding of the infant sera to RBD-coated ELISA plates pre- and post-infection with a XBB* variant

Sera binding to XBB.1.5 RBD-coated plates measured using enzyme-linked immunosorbent assay (ELISA). The low RBD binding activity in the sera collected prior to infection indicates maternal SARS-CoV-2 antibodies had decayed to very low levels prior to infection. As a negative control, we binding is also shown for a pool of human sera collected prior to the beginning of the COVID-19 pandemic. Only four of the 6 infants in the *primary XBB* infection infant sera* group had pre-infection sera available. The pre-infection sera was collected 11-46 days before infection and therefore at the time of infection maternal antibodies may have decayed further. See **Table S1** for details on sera collection times.

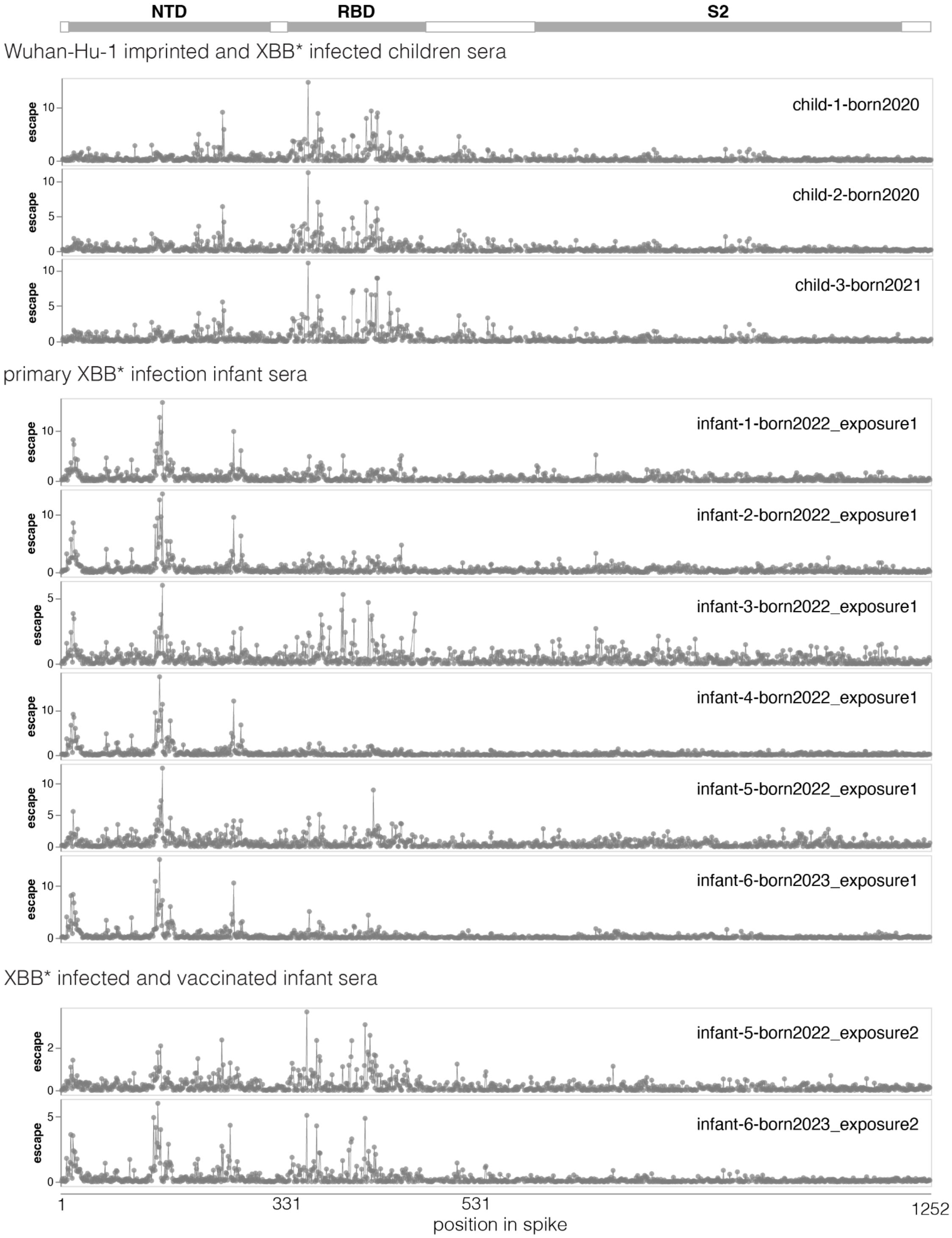

Supplementary Figure 2. Deep mutational scanning escape maps for individual sera

These plots are similar to those in **Fig. 2A** except they show escape for the individual sera in each set rather than the average across all sera in each set. This figure only shows the plots for the three sera sets that were newly characterized in this study; the plots for the *Wuhan-Hu-1-imprinted and XBB* infected adult sera* have been previously published in Dadonaite et al. 2024 (1). See

https://dms-vep.org/SARS-CoV-2_XBB.1.5_spike_DMS_infant_sera/individual.html for interactive versions of these plots for all four sera sets.

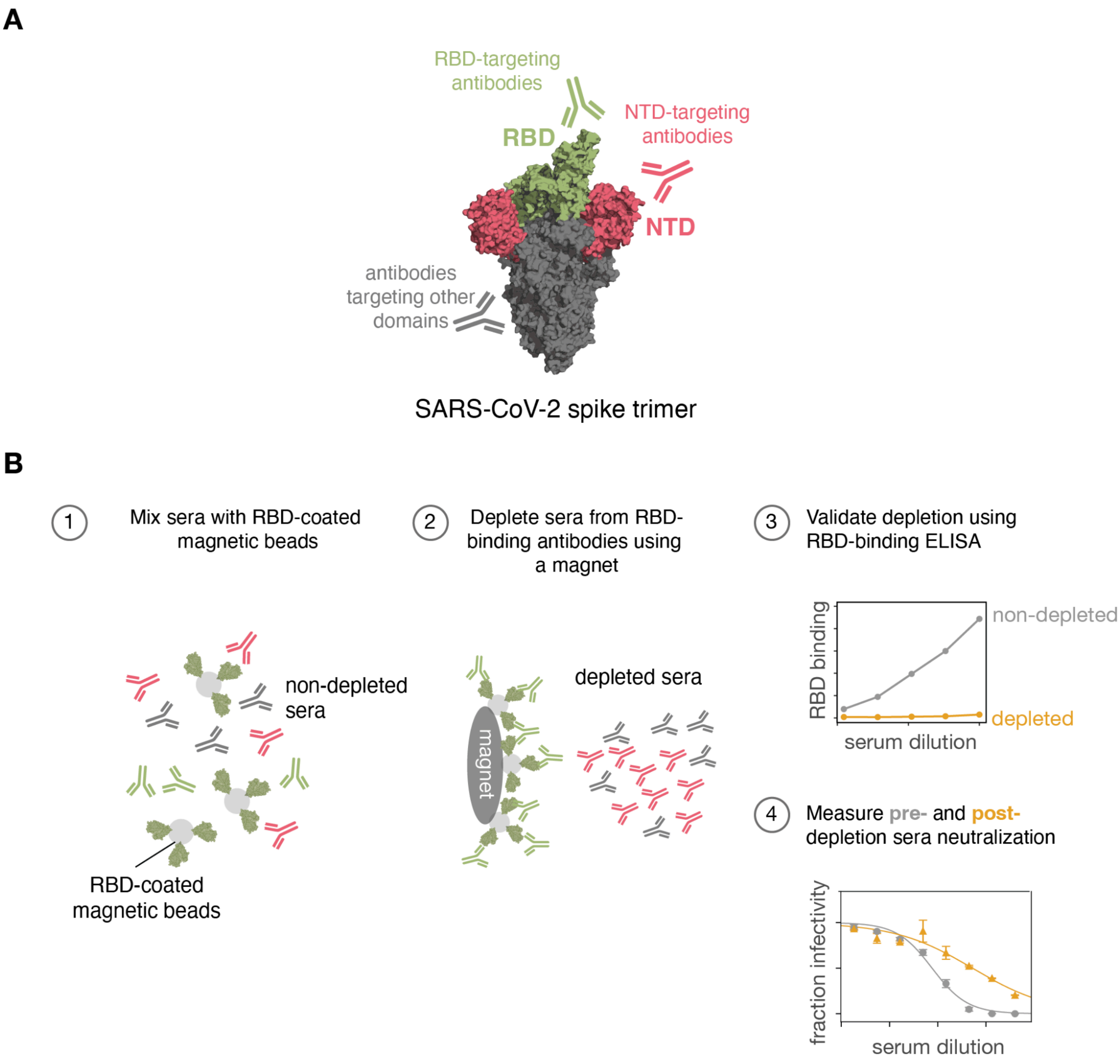

Supplementary Figure 3. Depletion of RBD-binding antibodies from sera to measure their contribution to neutralization

A. Schematic of SARS-CoV-2 spike trimer labeling different domains (PDB: 6XM4). **B.** Each serum was mixed with RBD coated magnetic beads to allow RBD-targeting antibodies to bind the beads. After binding, a magnet was used to deplete sera of the beads. To validate successful RBD-binding antibody depletion enzyme-linked immunosorbent assay (ELISA) was used. Depleted and non-depleted sera was used to perform pseudovirus neutralization assays.

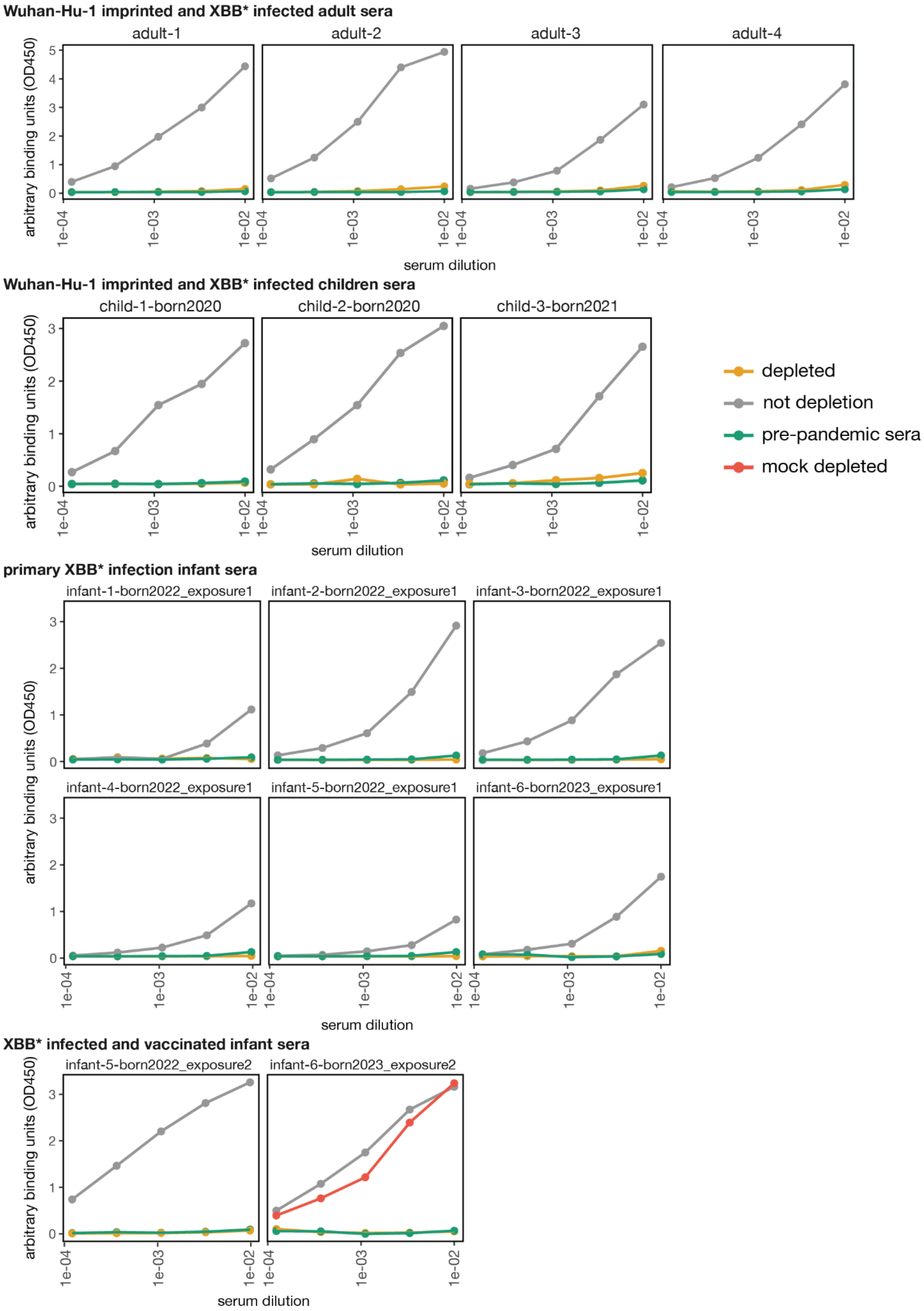

Supplementary Figure 4. Binding of sera to RBD-coated ELISA plates pre- and post-depletion of RBD-binding antibodies

Depleted and non-depleted sera binding to XBB.1.5 RBD-coated plates measured using enzyme-linked immunosorbent assay (ELISA). Serum depletion was performed as described in **Fig. S2**. Sera was depleted by multiple rounds of XBB.1.5 RBD-coated bead incubation until its binding was similar to the pre-pandemic sera (sera collected prior to 2020 that should not contain any antibodies specific to SARS-CoV-2). A mock depleted sample, which was incubated with beads lacking XBB.1.5 RBD, is shown for *infant-6-born2020_exposure2* serum.

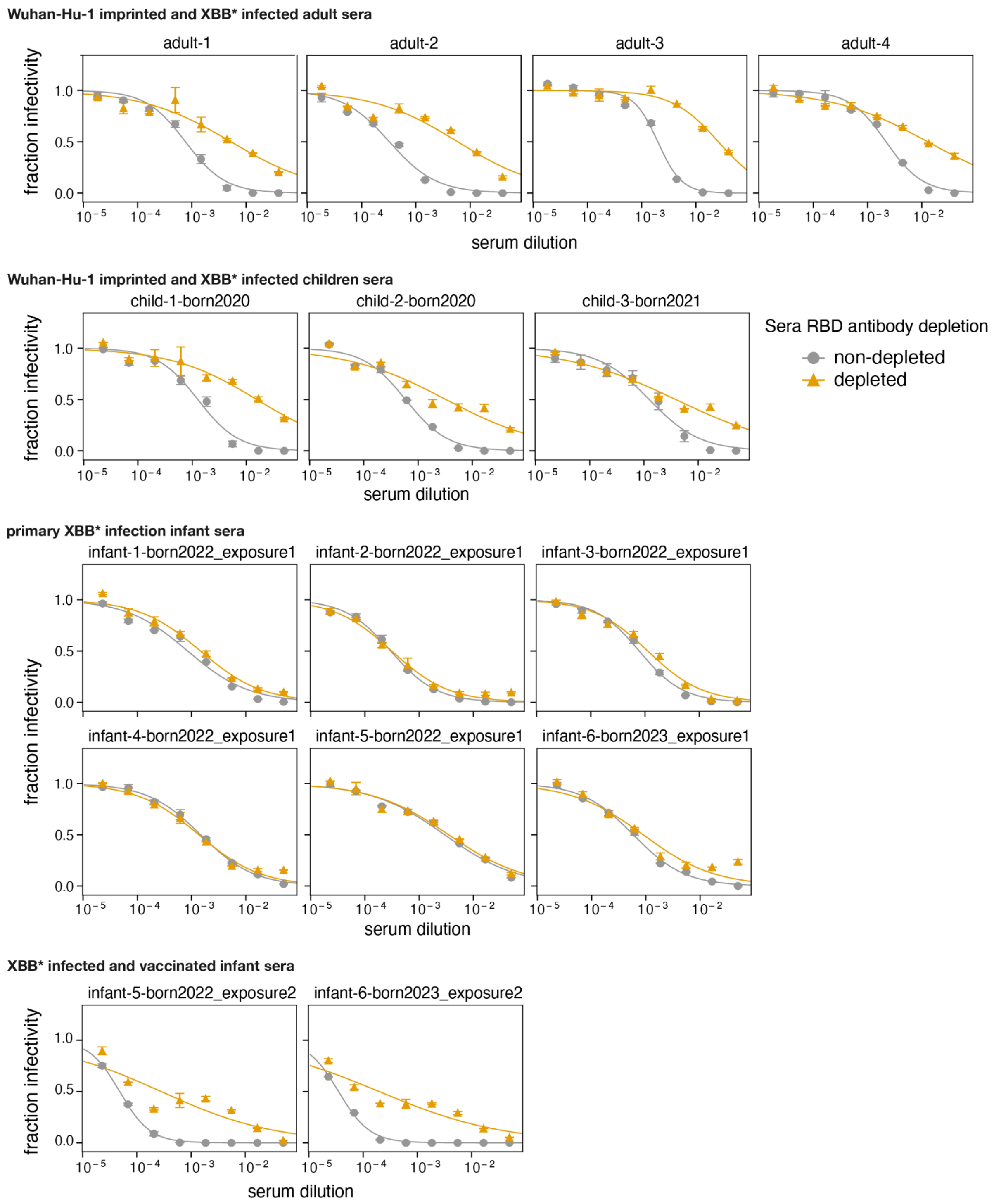

Supplementary Figure 5. Neutralization of XBB.1.5 pseudovirus pre- and post-depletion of RBD-binding antibodies

Neutralization curves for XBB.1.5 pseudovirus against depleted and non-depleted sera from **Fig. S3**. Neutralization assays were performed on 293T cells expressing medium levels of ACE2 as described in Farrell et al. 2022 (20). These target cells enable better detection of the neutralizing activity NTD-binding antibodies than standard 293T-ACE2 cells that express higher levels of ACE2.

NTD mutations

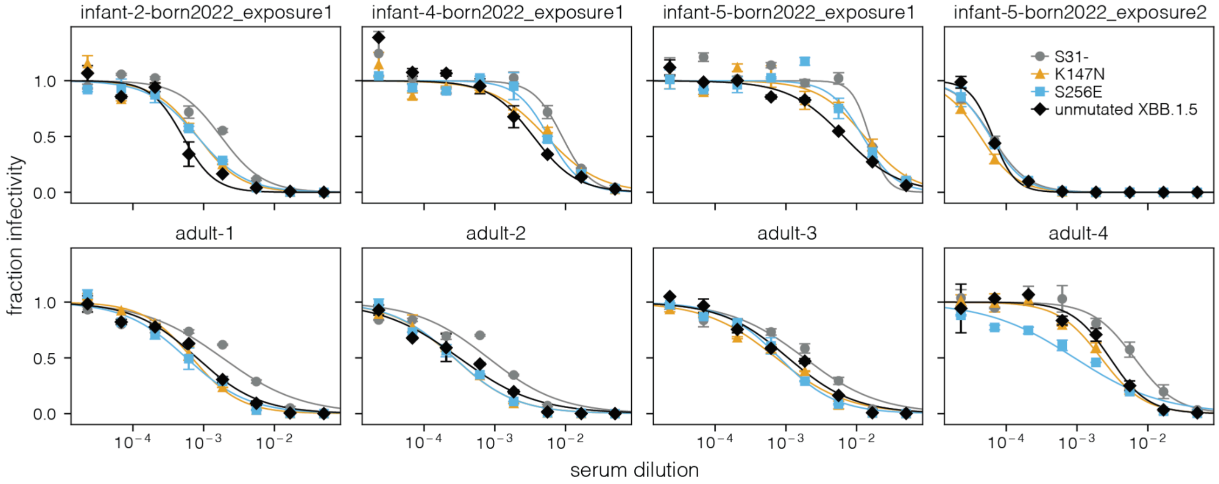

RBD mutations

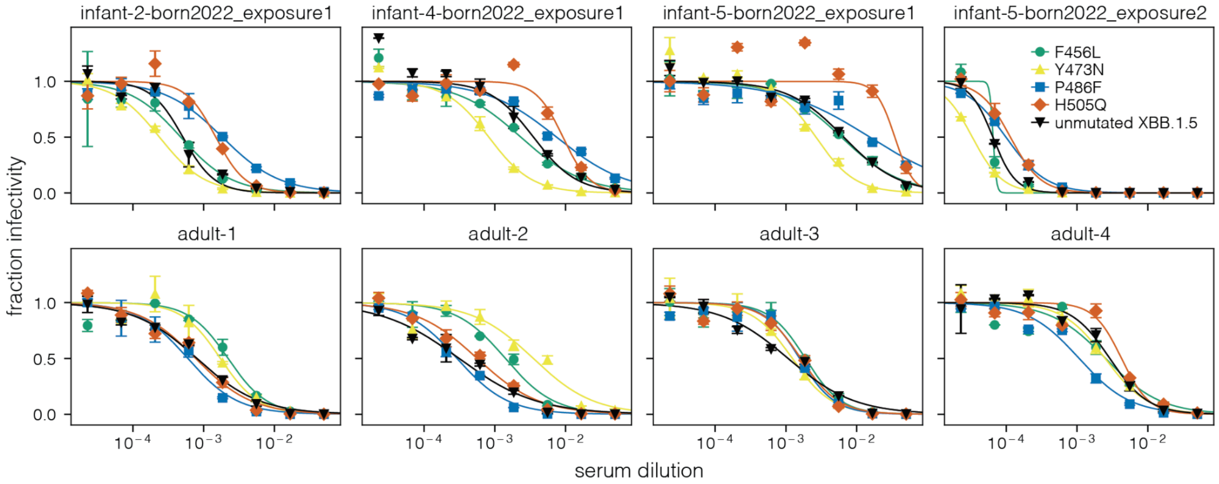

Supplementary Figure 6. Pseudovirus neutralization of different XBB.1.5 spike mutants
Neutralization curves for unmutated XBB.1.5 and mutant XBB.1.5 spike pseudoviruses against different sera. All mutants contain a single amino-acid change on the background of the XBB.1.5 spike.

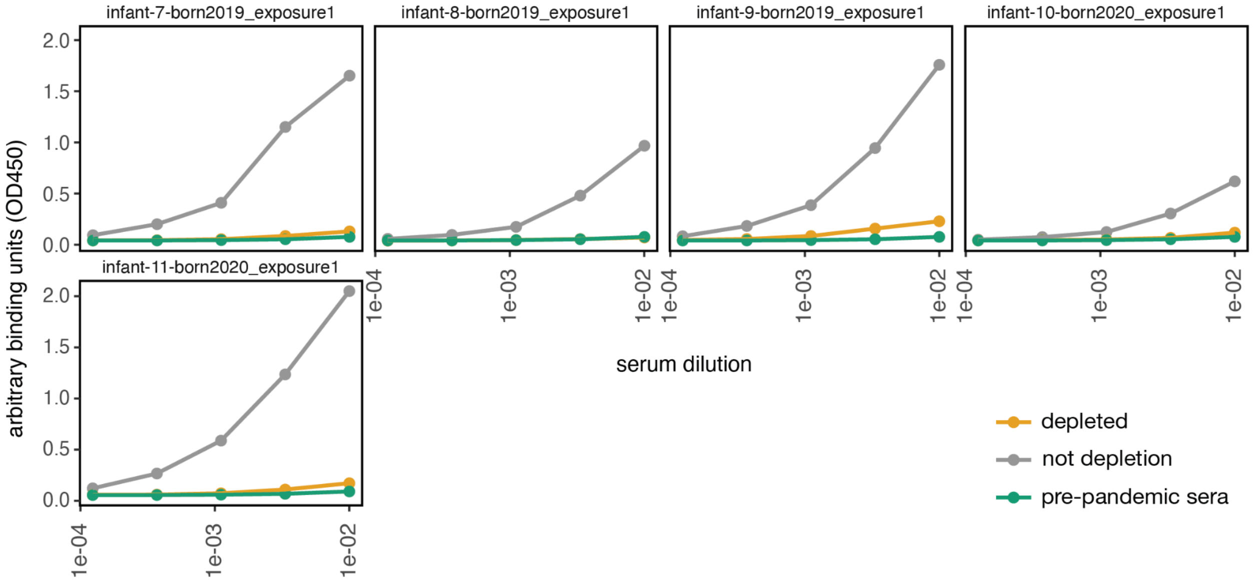

Supplementary Figure 7. Binding of sera to RBD-coated ELISA plates pre- and post-depletion of RBD-binding antibodies for primary Wuhan-Hu-1 infant infection sera
Depleted and non-depleted sera binding to Wuhan-Hu-1 RBD-coated plates measured using enzyme-linked immunosorbent assay (ELISA). Serum depletion was performed as described in **Fig. S2** except using beads conjugated to the Wuhan-Hu-1 RBD rather than the XBB.1.5 RBD. Sera was depleted in multiple rounds of RBD-coated bead incubation until its binding was similar to the pre-pandemic sera (sera collected prior to 2020 that should not contain any antibodies specific to SARS-CoV-2). Note that depletions for *primary Wuhan-Hu-1 infection adult sera* were done for a previous study and that same pre- and post-depletion sera was reused for this study; pre- and post-depletion ELISA binding data for that sera comparable to that shown here can be found in the associated Greaney et al. (2021) paper (3).

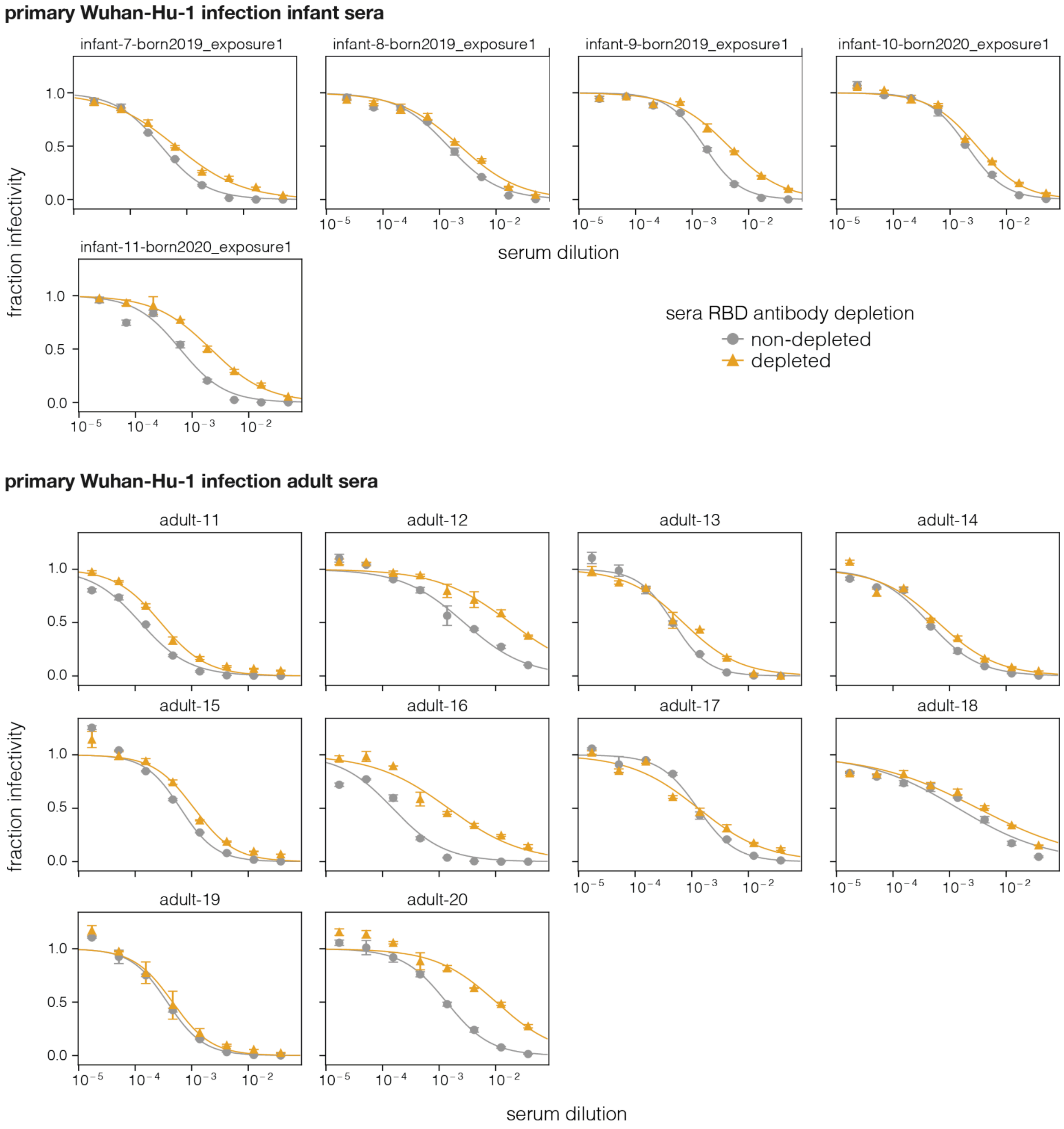

Supplementary Figure 8. Neutralization of D614G pseudovirus pre- and post-depletion of RBD-binding antibodies for primary Wuhan-Hu-1 infections

Neutralization curves for D614G pseudovirus against depleted and non-depleted primary Wuhan-Hu-1 infection infant and adult sera. Neutralization assays were performed on 293T cells expressing medium levels of ACE2 cells as described in Farrell et al. 2022 (2).

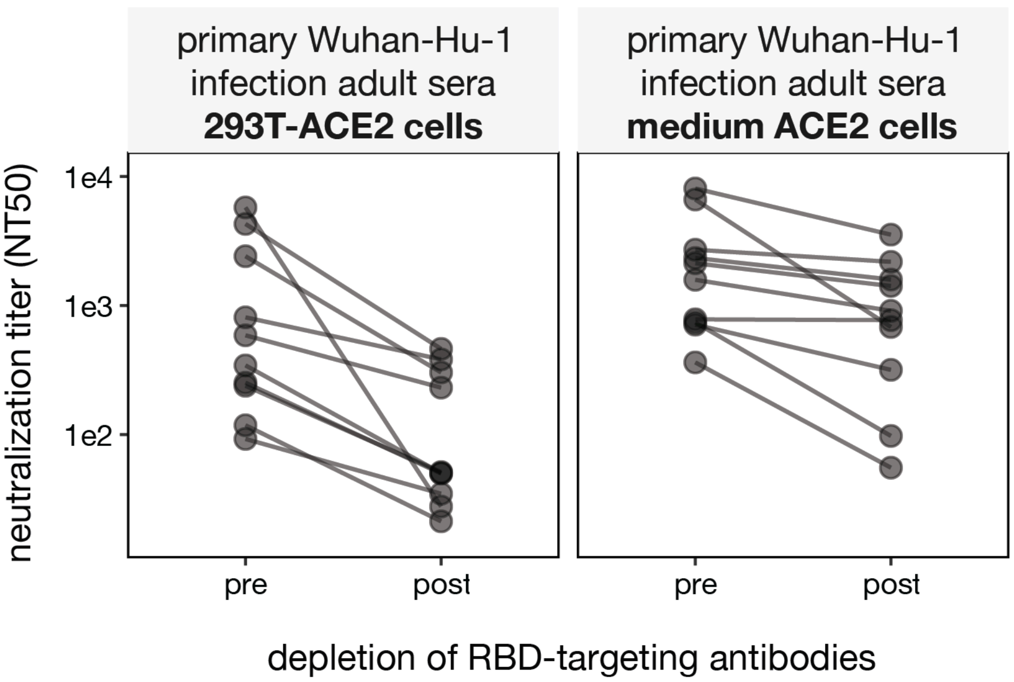

Supplementary Figure 9. The extent that serum neutralization is due to RBD-binding antibodies depends on the ACE2 expression of the target cells

Neutralization of pseudovirus expressing the D614G spike by *primary Wuhan-Hu-1 infection adult sera* pre- and post-depletion of Wuhan-Hu-1 RBD targeting antibodies as measured on two different target cell lines. The left panel shows data replotted from Greaney et al. 2021 (3) for neutralization assays performed using the 293T-ACE2 cells (4), which express high levels of ACE2. The right panel shows new measurements made using the same pre- and post-depletion serum samples but with the 293T cells expressing medium levels of ACE2 (2). These medium ACE2 cells express less ACE2 than the 293T-ACE2 cells; see first supplementary figure of Farrell et al. 2022 (2).

group name in this manuscript	sera sample name	spike exposure	sequence confirmed last infection variant	cohort	last exposure date	days post exposure	age at exposure	published previously	name in the previous study
primary XBB* infection infant sera	infant-1-born2022_exposure1	1x SARS-CoV-2 infection	XBB.1.5.16	IMPRINT	January-2023	27	7 mth		
	infant-2-born2022_exposure1	1x SARS-CoV-2 infection	XBB.1.16.1	IMPRINT	October-2023	23	11 mth		
	infant-3-born2022_exposure1	1x SARS-CoV-2 infection	JC.6	IMPRINT	July-2023	28	8 mth		
	infant-4-born2022_exposure1	1x SARS-CoV-2 infection	GK.2	IMPRINT	September-2023	37	9 mth		
	infant-5-born2022_exposure1	1x SARS-CoV-2 infection	FL.1.5.1	IMPRINT	September-2023	39	9 mth		
	infant-6-born2023_exposure1	1x SARS-CoV-2 infection	undetermined	IMPRINT	September-2023	44	7 mth		
Wuhan-Hu-1 imprinted and XBB* infected children sera	child-1-born2020	2x Wuhan-1 spike vaccination followed by 1x SARS-CoV-2 infection	XBB.1.5	IMPRINT	July-2022	82	22 mth		
	child-2-born2020	3x Wuhan-1 spike vaccination (3rd may have been bivalent vaccine) followed by 1x SARS-CoV-2 infection	XBB.1.16	IMPRINT	July-2022	72	14 mth		
	child-3-born2021	2x Wuhan-1 spike vaccination followed by 1x SARS-CoV-2 infection	XBB.1.5	IMPRINT	July-2022	63	13 mth		
Wuhan-Hu-1 imprinted and XBB* infected adult sera	adult-1	5x Wuhan-1 spike vaccination and 1x SARS-CoV-2 infections	XBB	HAARVI	January-2022	64	70 yrs	Dadonaite et al. 2024	498C
	adult-2	5x Wuhan-1 spike vaccination and 1x SARS-CoV-2 infections	no data	HAARVI	March-2023	24	63 yrs	Dadonaite et al. 2024	500C
	adult-3	5x Wuhan-1 spike vaccination and 1x SARS-CoV-2 infections	no data	HAARVI	March-2023	23	53 yrs	Dadonaite et al. 2024	501C
	adult-4	3x Wuhan-1 spike vaccination and 1x SARS-CoV-2 infections	XBB.1.5	HAARVI	February-2023	89	41 yrs	Dadonaite et al. 2024	505C
	adult-5	5x Wuhan-1 spike vaccination and 3x SARS-CoV-2 infections	no data	HAARVI	March-2023	73	57 yrs	Dadonaite et al. 2024	287C
	adult-6	5x Wuhan-1 spike vaccination and 3x SARS-CoV-2 infections	no data	HAARVI	March-2023	74	56 yrs	Dadonaite et al. 2024	288C
	adult-7	4x Wuhan-1 spike vaccination and 1x SARS-CoV-2 infections	no data	HAARVI	April-2023	31	19 yrs	Dadonaite et al. 2024	343C
	adult-8	2x Wuhan-1 spike vaccination and 1x SARS-CoV-2 infections	XBB.1.5	HAARVI	January-2023	26	28 yrs	Dadonaite et al. 2024	493C
	adult-9	4x Wuhan-1 spike vaccination and 2x SARS-CoV-2 infections	no data	HAARVI	March-2023	18	48 yrs	Dadonaite et al. 2024	497C
	adult-10	6x Wuhan-1 spike vaccination and 1x SARS-CoV-2 infections	no data	HAARVI	May-2023	15	51 yrs	Dadonaite et al. 2024	503C

**XBB* infected and vaccinated infant sera**	infant-5-born2022_exposure2	SARS-CoV-2 infection followed by XBB15 spike vaccination (based on the date vaccine was given)		IMPRINT	November-2023	35	12 mth		
	infant-6-born2023_exposure2	SARS-CoV-2 infection followed by XBB15 spike vaccination (based on the date vaccine was given)		IMPRINT	December-2023	26	10 mth		
**primary Wuhan-Hu-1 infection infant sera**	infant-7-born2019_exposure1	1x SARS-CoV-2 infection	B.1.2	IMPRINT	January-2021	70	13 mth		
	infant-8-born2019_exposure1	1x SARS-CoV-2 infection	B.1	IMPRINT	November-2020	23	12 mth		
	infant-9-born2019_exposure1	1x SARS-CoV-2 infection	B.1.2	Immune Response	November-2020	51	12 mth		
	infant-10-born2020_exposure1	1x SARS-CoV-2 infection	B.1	IMPRINT	December-2020	28	7 mth		
	infant-11-born2020_exposure1	1x SARS-CoV-2 infection	B.1	IMPRINT	March-2021	84	8 mth		
**primary Wuhan-Hu-1 infection adult sera**	adult-11	1x SARS-CoV-2 infection	no data	HAARVI	March-2020	45	56 yrs	Greaney et al. 2021	subject A
	adult-12	1x SARS-CoV-2 infection	no data	HAARVI	March-2020	26	65 yrs	Greaney et al. 2021	subject I
	adult-13	1x SARS-CoV-2 infection	no data	HAARVI	March-2020	26	56 yrs	Greaney et al. 2021	subject M
	adult-14	1x SARS-CoV-2 infection	no data	HAARVI	March-2020	28	65 yrs	Greaney et al. 2021	subject E
	adult-15	1x SARS-CoV-2 infection	no data	HAARVI	March-2020	29	65 yrs	Greaney et al. 2021	subject K
	adult-16	1x SARS-CoV-2 infection	no data	HAARVI	March-2020	32	76 yrs	Greaney et al. 2021	subject C
	adult-17	1x SARS-CoV-2 infection	no data	HAARVI	March-2020	32	29 yrs	Greaney et al. 2021	subject P
	adult-18	1x SARS-CoV-2 infection	no data	HAARVI	March-2020	32	23 yrs	Greaney et al. 2021	subject N
	adult-19	1x SARS-CoV-2 infection	no data	HAARVI	February-2020	33	64 yrs	Greaney et al. 2021	subject D
	adult-20	1x SARS-CoV-2 infection	no data	HAARVI	March-2020	35	68 yrs	Greaney et al. 2021	subject L
**infant sera before XBB* infection**	infant-2-born2022_preinfection	sera collected before infection, not exposed to spike	NA	IMPRINT	October-2023	-46			
	infant-4-born2022_preinfection	sera collected before infection, not exposed to spike	NA	IMPRINT	September-2023	-19			

	infant-5-born2022_pre infection	sera collected before infection, not exposed to spike	NA	IMPRINT	September-2023	-24			
	infant-6-born2023_pre infection	sera collected before infection, not exposed to spike	NA	IMPRINT	September-2023	-11			

Supplementary Table 1. Details of the individual sera used in this study

Individual sera from each cohort used in this study. *Primary Wuhan-Hu-1 infected adult sera* and some of the *Wuhan-Hu-1 imprinted and XBB* infected adult sera* do not have sequencing data available for infections, however, based on infection dates the former cohort is most likely exposed to early strains closely related to Wuhan-Hu-1, and the latter to XBB*-related variants. The nasal swab from *infant-6-born2023_exposure1* did not yield sufficient coverage to confirm infecting variant but based on the date exposed this was most likely XBB* related variant. Negative values in the days post-exposure column indicate the number of days before exposure the sera were collected.

References

1. Dadonaite B, Brown J, McMahon TE, Farrell AG, Figgins MD, Asarnow D, et al. Spike deep mutational scanning helps predict success of SARS-CoV-2 clades. Nature. 2024 Jul;631(8021):617–26.
2. Farrell AG, Dadonaite B, Greaney AJ, Eguia R, Loes AN, Franko NM, et al. Receptor-Binding Domain (RBD) Antibodies Contribute More to SARS-CoV-2 Neutralization When Target Cells Express High Levels of ACE2. Viruses. 2022 Sep;14(9):2061.
3. Greaney AJ, Loes AN, Crawford KHD, Starr TN, Malone KD, Chu HY, et al. Comprehensive mapping of mutations in the SARS-CoV-2 receptor-binding domain that affect recognition by polyclonal human plasma antibodies. Cell Host Microbe. 2021 Mar 10;29(3):463-476.e6.
4. Crawford KHD, Eguia R, Dingens AS, Loes AN, Malone KD, Wolf CR, et al. Protocol and Reagents for Pseudotyping Lentiviral Particles with SARS-CoV-2 Spike Protein for Neutralization Assays. Viruses. 2020 May 6;12(5):513.
